# Chipster: user-friendly analysis software for microarray and other high-throughput data

**DOI:** 10.1186/1471-2164-12-507

**Published:** 2011-10-14

**Authors:** M Aleksi Kallio, Jarno T Tuimala, Taavi Hupponen, Petri Klemelä, Massimiliano Gentile, Ilari Scheinin, Mikko Koski, Janne Käki, Eija I Korpelainen

**Affiliations:** 1 CSC - IT Center for Science, Keilaranta 14, Keilaniemi, Espoo, Finland; 2 Finnish Red Cross Blood Service, Kivihaantie 7, Helsinki, Finland; 3 Department of Pathology, VU University Medical Center, Amsterdam, The Netherlands; 4 Department of Pathology, Haartman Institute and HUSLAB, University of Helsinki and Helsinki University Central Hospital, Finland; 5 FIMM Technology Centre, Institute for Molecular Medicine Finland (FIMM), University of Helsinki, Finland; 6 Futurice, Vattuniemenranta 2, Helsinki, Finland

## Abstract

**Background:**

The growth of high-throughput technologies such as microarrays and next generation sequencing has been accompanied by active research in data analysis methodology, producing new analysis methods at a rapid pace. While most of the newly developed methods are freely available, their use requires substantial computational skills. In order to enable non-programming biologists to benefit from the method development in a timely manner, we have created the Chipster software.

**Results:**

Chipster (http://chipster.csc.fi/) brings a powerful collection of data analysis methods within the reach of bioscientists via its intuitive graphical user interface. Users can analyze and integrate different data types such as gene expression, miRNA and aCGH. The analysis functionality is complemented with rich interactive visualizations, allowing users to select datapoints and create new gene lists based on these selections. Importantly, users can save the performed analysis steps as reusable, automatic workflows, which can also be shared with other users. Being a versatile and easily extendable platform, Chipster can be used for microarray, proteomics and sequencing data. In this article we describe its comprehensive collection of analysis and visualization tools for microarray data using three case studies.

**Conclusions:**

Chipster is a user-friendly analysis software for high-throughput data. Its intuitive graphical user interface enables biologists to access a powerful collection of data analysis and integration tools, and to visualize data interactively. Users can collaborate by sharing analysis sessions and workflows. Chipster is open source, and the server installation package is freely available.

## Background

The growth of high-throughput technologies such as microarrays and next generation sequencing (NGS) has been accompanied by active research in data analysis methodology, producing new analysis methods at a rapid pace. The international Bioconductor project [1] has been particularly important in this regard, demonstrating the power of open software development for bioinformatics. While most of the newly developed methods are freely available, their use requires substantial computational skills, such as knowledge of the R programming language in the case of Bioconductor. This can be a bottleneck for wet lab scientists, who typically have a life science background and no programming experience. In order to enable experimental biologists to benefit from the method development in a timely manner, we have created the Chipster software [2]. Chipster brings a powerful collection of up-to-date analysis methods and visualization tools within the reach of bioscientists via its intuitive graphical user interface. Being a versatile and easily extendable platform, Chipster can be used for different types of high-throughput data such as microarrays, proteomics and NGS. In this article we describe its comprehensive collection of analysis and visualization tools for microarray data using three case studies.

## Implementation

Chipster's ability to provide a biologist-friendly access to a powerful bioinformatics platform is technically based on a desktop application user interface, a flexible distributed architecture, and the ability to integrate many types of analysis tools.

The Chipster client software is a full graphical Java desktop application, since we saw it the best way for offering an intuitive user interface with highly interactive visualisations and an overall smooth user experience. To make the client installation and updates as easy and automatic as possible, Chipster uses the Java Web Start technology.

In order to provide a comprehensive set of analysis tools, we have made it easy to integrate any kind of tools in Chipster, regardless of how they are implemented (R/Bioconductor, command line, Java, Web services, etc.). As R/Bioconductor provides a rich collection of analysis functionality for microarray and NGS data, we have built a strong support for R integration: Wrappers manage communication with R processes and pool them for rapid responsiveness, and several R versions can be run side-by-side. Integration of command line tools is also supported and can be accomplished even automatically. The tool selection offered by the local server can be augmented by external Web services (SOAP). For example, we currently use the pathway analysis tools for IntAct, Reactome and ConsensusPathDB in this manner. From the user's point of view these remote services look like any other tools and are included in the workflows as usual.

**Figure 1:**
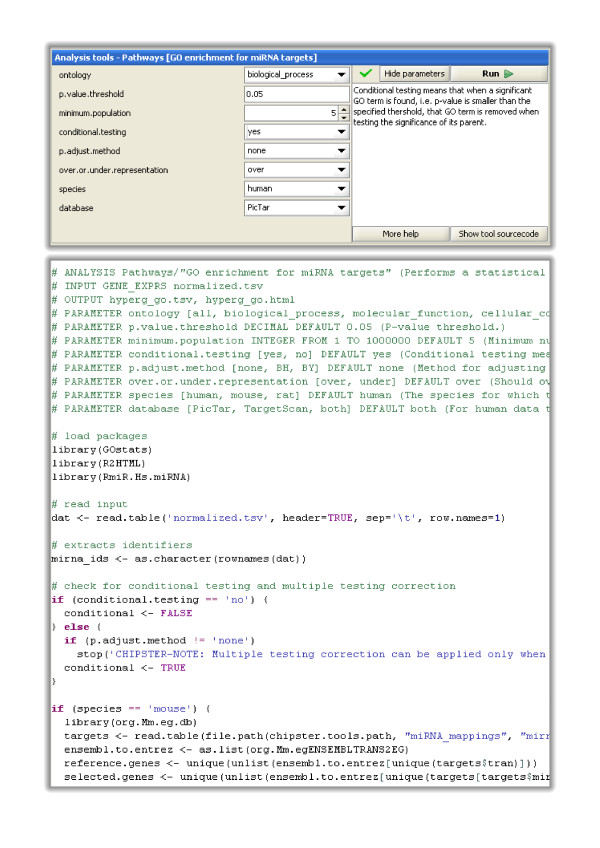
**Analysis tools are integrated to Chipster using a simple tool description notation**. It is easy to integrate new analysis tools to Chipster. One simply writes a short description for the tool's inputs, outputs and parameters as shown here. When the description and the tool code are placed to a specific directory in the computing service, the tool and its parameter panel become automatically visible to the users in the Chipster client program. This example shows the automatically generated parameter panel (top) and the description and tool code (below) for the R/Bioconductor based tool "Pathway analysis for miRNA targets". In the case of a command line tool, instead of copying the tool code, one just adds a reference to the tool binary in the Chipster configuration file.

Adding new tools to Chipster is easy. First you write a short description for the tool's inputs, outputs and parameters using simple notations (Figure 1). Then you place the description and the tool code, for example an R script, to a specific directory in the computing service. In the case of a command line tool, instead of copying the tool code, you just add a reference to the tool binary in the Chipster configuration file. The new tool is then picked up by the computing service and becomes automatically visible in the Chipster client.

In the basic setup, Chipster is a client-server system. Server architecture allows tasks to be performed in optimal places: for example, interactive visualizations happen in the client, whereas the actual analysis tasks are processed by computing services, which can be run on server machines with ample CPU and memory resources. This way the user can run several analysis tasks simultaneously without burdening his/her computer. In addition, there is no need to install any analysis tools or libraries to the user's computer as they are installed and maintained centrally in the computing servers. To avoid transferring data multiple times between the client and server, a caching mechanism is used. The caching extends to multi-user scenarios thanks to Chipster's cryptographically strong data identifiers: When a previously saved analysis session is opened from a different computer, possibly by a different user, the system still uses the original cached copy of the data and does not transfer it again to the server side.

A Chipster server can be run on a single server computer or even a laptop. The Chipster server itself contains multiple independent services, so it can be scaled across a cluster of servers to distribute computational and data transfer load. Reliability is also improved as failed services can be replaced on the fly. The runtime scaling has proven to be useful when operating large national and institutional Chipster services, as usage peaks can be managed by adding computational servers when required. The system consists of compute, authentication and management services, and message and file brokers, which act as the communication channels between the components (see additional file 1: Chipster_architecture.pdf). Because of the message oriented architecture, only the broker components require open network ports and therefore local firewalls should not pose problems. Only the message broker needs to be configured as an entry point into the system, all the other server components are automatically discovered. For running the computing services, a 64-bit Linux or Mac computer is recommended. The other server parts and the client software only require Java 1.6.

Chipster is freely available and is open source software under the GNU General Public License (GPL) version 3 or later. We provide an installation package for the software of the complete Chipster server system at the Sourceforge site [3], and installation instructions for this package can be found on our Wiki pages [4]. Free short-term evaluation accounts to our Chipster server are provided for those wanting to try Chipster first, and long term accounts are also available [2]. Installation of the basic server setup is straightforward using the tools provided, and the Wiki pages also document the more advanced adaptations, such as distribution to a cluster, integration into local authentication systems, and deployment of secure communication protocols. After unpacking the server software and running an automatic configuration script, the server can be started and the Chipster client launched via a web site provided with the package. All analysis tools are included in the installation by default, but most of them require R or other supporting applications to function. A setup script is provided that automatically installs the R packages, and instructs how the supporting applications should be installed. The server administrator is free to tailor the tool selection and install tool support only for those functionalities that are needed. For even more rapid deployment of the Chipster server environment, we are developing a virtual machine based package.

## Results

### General functionality of Chipster

#### User interface

**Figure 2:**
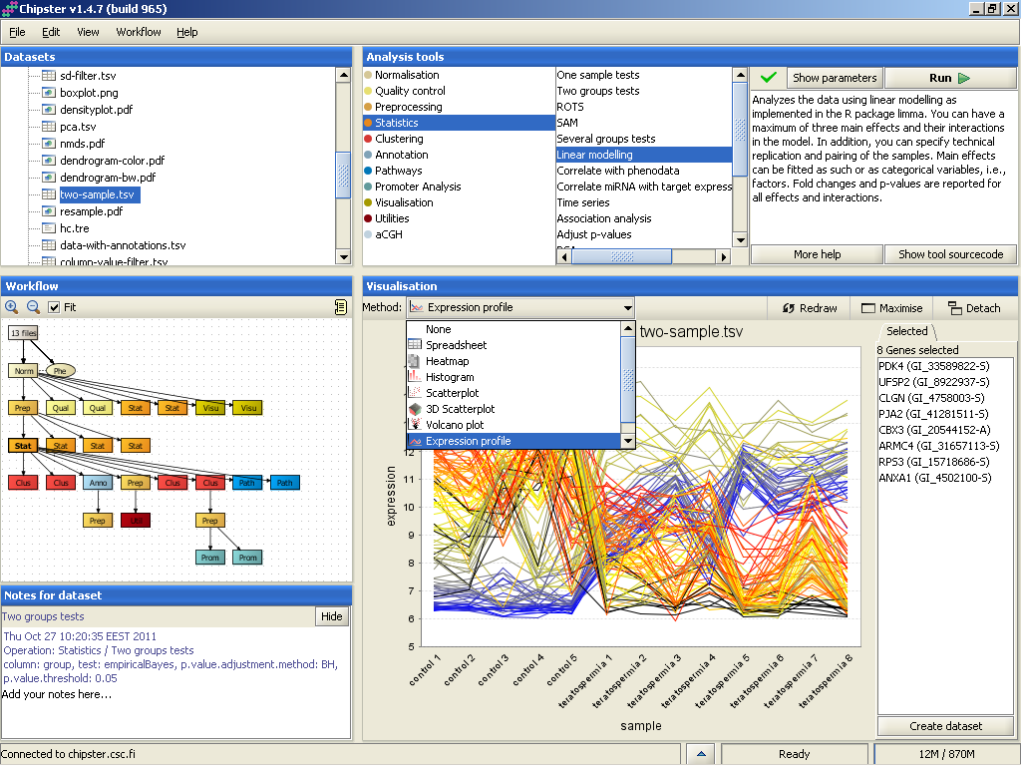
**Chipster user interface**. Chipster's user interface consists of four panels: Datasets, analysis tools, workflow, and visualization. Data files are displayed in the Datasets view (top left) and in the Workflow view (bottom left). The latter displays the analysis results as boxes colored according to the category of the tool that produced them. It enables the user to keep track of the relationships of the result files and to save the analysis steps taken as an automatic, reusable workflow. The analysis tool panel (top right) displays Chipster's analysis tools grouped into categories such as normalization, preprocessing, statistics and pathway analysis for easy discovery. Once a tool has been selected, the user can view its short description, the manual page and the source code, and change parameters if necessary. The visualization panel (bottom right) offers different interactive visualizations according to the selected dataset. The user can select genes in the visualizations and create new datasets based on this selection.

Chipster's user interface consists of four panels: Analysis tools, datasets, workflow and visualization (Figure 2). The panels for the datasets and the workflow display essentially the same files, but while the former provides a typical folder view, the latter shows the relationships between the files. It is therefore easy to keep track of which analysis steps were taken to produce a particular file. Both views allow the user to export, rename, and delete files, and the workflow view also allows the user to prune and save workflows. The analysis tool panel displays Chipster's analysis tools grouped into categories such as normalization, preprocessing, statistics and pathway analysis for easy discovery. Once a tool has been selected, the user can view its short description, the manual page and the source code, and change parameters if necessary. A complete list of the current analysis tools is available on the Chipster web site, and the analysis functionality is described in more detail in the corresponding section of this article. The visualization panel allows the user to view the selected dataset using different visualization methods, which are discussed in more detail below.

Describing an experimental setup is accomplished using a Phenodata editor. Chipster's normalization tools produce a phenodata file, which the user can complete by entering the experimental groups for the different samples. Any other variables such as time, dose, pairing and technical replicates can also be entered by adding new columns to the phenodata. The description column allows the user to enter the sample names that s/he wants to be used in visualizations. Phenodata is by default created during normalization, but users can also import normalized data and generate a phenodata file for it in Chipster, as demonstrated in the second case study of this article.

When an analysis task has been submitted, its progress can be monitored by opening the Task manager window from the bottom panel of the user interface. Task manager lists the status (i.e. transferring inputs, waiting, running, transferring outputs, completed), starting and running times, and tool parameters. It also allows the user to cancel a task if needed.

Chipster allows users to save their analysis sessions, so that the work can be continued later, even on another computer, or shared with collaborators. Work on different datasets can be saved into separate sessions, and the sessions can also be combined later if needed. A session file is a zip-file containing all the data files, their relationships, and the tool parameters used for each analysis step. It is also possible to save just the commands for the analysis steps taken as a workflow, which can be applied to another dataset or shared with other users. The workflow functionality of Chipster is described in more detail later in this article.

A complete manual for Chipster describing data import, user interface and the individual analysis tools is available on the web [2]. It also contains step-by-step tutorials which cover whole analysis from data import to downstream applications such as pathway enrichment using publicly available datasets. While helpful for individual users getting started with Chipster, the tutorials can also serve as teaching material in microarray data analysis courses. Several Chipster training sessions are organized every year in different locations, the details can be found on Chipster website.

#### Visualizations

Visualizing data and inspecting it by eye is one of the most powerful ways of finding patterns that are interesting for further analysis. We have therefore made a lot of effort to provide rich and powerful visualizations in Chipster. Currently there are about 25 different visualizations, which are divided in two categories: interactive visualizations generated by the client program, and static images generated by R/Bioconductor on the server. Both types of visualizations are viewed in the visualization panel (Figure 2). This panel can be maximized if more area is required for viewing, or detached as a separate window if several visualizations need to be viewed simultaneously.

Chipster's interactive visualizations include 2D and 3D scatter plots, histogram, expression profiles, array layout, volcano plot, Venn diagram, heatmap and self-organizing map clustering (SOM) visualization. In addition to zooming and changing titles and colors etc, the interactive visualizations allow users to select datapoints and create new gene lists based on these selections. There is cross-talk between the different visualization methods, so that datapoints selected in one visualization are highlighted when the same data is visualized using another method. All interactive visualizations can be saved in PNG format by right-clicking on the image.

R/Bioconductor provides a wide variety of visualizations for microarray data, many of which are available in Chipster. These include box plot, density plot, heatmap, correlogram, annotated dendrogram, MA plot, idiogram, quality control plots, gene set enrichment plots, and several visualizations for array comparative genomic hybridization (aCGH) data. As opposed to the interactive visualizations generated by the Chipster client program, the images generated by R/Bioconductor are static, although in many of them the user can change the sample names by entering the desired names in the phenodata file as described above.

#### Automatic workflows speed up analysis and enable reproducible and collaborative research

Microarray data analysis typically involves performing several analysis steps and trying different parameter settings. Once a suitable combination has been found and analysis completed, it is often desirable to save the steps taken as an automatic workflow. Reusing workflows serves many purposes. Firstly, it saves time as multi-step analysis can be executed with just one mouse click. Sharing workflows within a research group brings consistency to analysis and provides an easy way for bioinformaticians to help biologists. Sharing workflows in a wider context is also beneficial as providing a downloadable workflow file facilitates the reproduction of published results and increases the collaboration of the bioinformatics community in general.

The need for automatic workflows is widely recognized and many programs such as GenePattern, Taverna and Galaxy [5-7] provide different approaches towards this goal, ranging from pure workflow enactment engines to analysis software with web forms for workflow construction. In Chipster we have taken an approach where, instead of specifically constructing workflows, the user performs the analysis normally. The system keeps track of the analysis steps taken, and displays them visually in the Workflow panel (Figure 2). The user can experiment with different methods and parameters, and prune the resulting workflow by deleting the unwanted steps. When a satisfactory analysis pipeline is ready, the user simply clicks on the desired beginning point of it in the workflow panel and saves the workflow. The workflow is saved as a file, which contains instructions to run certain analysis tools with the selected parameter settings in a certain order. Importantly, Chipster also supports branched workflows, as real life analysis workflows are seldom simple linear sequences of steps.

Users can easily apply a workflow to another dataset, or share it with other Chipster users by giving them a copy of the workflow file. In addition to the user-made workflows, Chipster provides ready-made workflows for finding and analyzing differentially expressed genes, miRNAs and proteins. The user can continue analysis from the workflow results as normal, so they don't restrict the analysis in any way but can be used rather as a backbone.

The primary goal of Chipster's workflow functionality is to enable non-programming users to construct workflows. However, users with programming experience can extend the Java BeanShell code of a workflow file with any functionality desired: the workflow environment is a complete programming environment and the functionality of the client can be accessed using a workflow programming interface.

### Analysis functionality

#### Data import and supported array types

Chipster is able to import any tab-delimited data. While Affymetrix CEL-files and Illumina BeadStudio/GenomeStudio-files are recognized automatically, other files are imported using an Import tool, which allows the user to specify the data columns corresponding to identifiers, sample and background intensities, etc. Chipster offers the possibility to import data not only from user's computer, but also directly from public databases such as ArrayExpress [8], Gene Expression Omnibus (GEO) [9], and CanGEM [10], and from a given url.

It is important to note that while the tools for preprocessing, statistics, clustering and visualizations work for any tab-delimited data, tools for annotation, pathway and promoter analysis require annotation information for the array. Chipster has annotation packages for most Affymetrix expression arrays (3', gene and exon arrays), all Illumina expression arrays and the human 27 k methylation array, and the most common Agilent expression arrays. In addition, rudimentary support is offered for Affymetrix and Illumina SNP arrays. For aCGH arrays it is essential to know the exact genomic coordinates for the probes, and Chipster has a dedicated tool for fetching these annotations from the CanGEM database [10]. For a full list of supported array types, please see the website [2]. Annotation packages for new arrays can be created using the AnnotationDbi package offered in the Bioconductor project.

#### Normalization

Chipster is capable of normalizing most of the commonly used chip types. It has dedicated normalization tools for Affymetrix 3', gene and exon arrays, Illumina arrays, and Agilent 1- and 2-color arrays. Chipster also offers a general normalization tool for cDNA arrays that can be used for normalizing other 2-color data. Similarly, the Agilent 1-color tool can be used for normalizing other 1-color data. The actual normalization methods, such as Robust Multi-array Average (RMA), Li-Wong (dChip), loess, quantile, robust spline and variance stabilizing normalization, are implemented as parameters of the tools [11,12].

It has been shown that a significant number of probes on several Affymetrix and Illumina arrays map to different genes than indicated by the manufacturer [13-16]. As remapping probes to the current genome and transcriptome databases has been shown to improve the interpretation of gene expression data, Chipster's normalization tools offer the possibility to use the remapped information. For Affymetrix' 3'-expression arrays the user can decide whether to use the alternative mappings (altCDFs) in the summarization step. For Affymetrix exon and gene arrays and for Illumina arrays the remappings are used automatically. The first case study of this article demonstrates how to apply the alternative mappings for Affymetrix' 3'-expression arrays.

After the initial normalization using a platform-specific tool, the data can be further normalized to specific genes or samples. Chipster also includes a tool for removing random (batch) effects, e.g. where samples cluster according to preparation day instead of the biological groups under study, using a linear mixed modelling approach to the normalization.

#### Quality control

Chipster has an extensive selection of tools for quality control. These include platform-specific tools, such as plots for RNA degradation, Relative Log Expression (RLE), Normalized Unscaled Standard Error (NUSE), scaling factor summary, percent of present probesets, and quality control probe expression in the case of Affymetrix arrays. The more general tools, such as Principal Component Analysis (PCA), clustering and Non-metric Multi-Dimensional Scaling (NMDS), can also be used for quality control of samples. If quality control tools indicate that certain samples need to be excluded from further analysis, this can be easily accomplished in Chipster by either excluding the deviant samples from the already normalized data or by re-normalizing the acceptable samples. The latter approach is recommended for certain normalization methods such as RMA, which are affected by the context (i.e. a set of arrays).

#### Filtering

Chipster includes tools for filtering genes by standard deviation, coefficient of variation, inter-quartile range, expression and flags. Another, more versatile way of filtering is to first calculate several descriptive statistics for each gene by using the specific tool for that, and then apply the "Filter using a column value" tool to filter the genes based on any of these. Annotated gene lists can also be filtered based on chromosomal location, pathway terms, etc. Different filters can be combined by using the interactive Venn diagram to create new subsets. Venn diagram can also be used for filtering the dataset with a list of gene identifiers.

#### Statistical testing

Statistical tools in Chipster can be divided into tests for finding differentially expressed genes, ordination methods and association analysis. Tools for pathway analysis as well as the statistical tools dedicated for aCGH data are described in their own sections below.

Tests for finding differences in mean gene expression between groups are divided into separate tools according to the number of groups to be compared (one group, two groups, several groups). Several tests are available in every tool, and they usually include both parametric tests such as t-test, empirical Bayes [17], ANOVA, and non-parametric tests such as Mann-Whitney U and Kruskall-Wallis' test. Chipster also contains separate tools for Significance Analysis of Microarrays (SAM) [18] and Reproducibility-Optimized Test Statistic (ROTS) [19]. A linear modelling tool, an implementation of linear regression modelling, allows analysis of several variables at the same time. It can take into account three main effects and their interactions, as well as technical replicates and pairing, and its use is demonstrated in the first case study of this article.

Ordination methods include PCA, NMDS, and Canonical Correspondence Analysis (CCA). PCA can be performed for either genes or samples, and the results can be visualized as an interactive 3D-scatter plot, where samples can be colored according to any experimental variable defined in the phenodata file.

Association analysis can perform case-control analyses on SNP array data. It tests Hardy-Weinberg equilibrium, and association of the genetic markers with the case-control status using both dominant and recessive models of inheritance.

#### Unsupervised and supervised clustering

Chipster's tools for unsupervised clustering include K-means, hierarchical and quality threshold clustering and SOM. Hierarchical clustering results can be visualized as interactive heatmaps and plain trees, and the reliability can be checked using bootstrapping. For K-means clustering, Chipster includes a separate tool for estimating the optimal number of clusters to generate (K).

Classification or supervised clustering tools include K-nearest neighbor (KNN)-classification and the more versatile general classification. KNN-classification allows validation of classifiers by using either a cross-validation approach or a test set of new samples. The general classification tool offers many more classification methods, such as Support Vector Machines (SVM), Linear Discriminant Analysis (LDA), and Naïve Bayes networks, but it does not allow classifying new samples like the KNN-classification does.

#### Annotation

Chipster uses annotation packages provided by the Bioconductor project and the BrainArray site [20]. There are two ways to annotate the data: either by generating a separate annotation file or by appending the annotation to the actual data. This latter option allows for filtering genes based on pathway involvement, chromosomal location, or other annotation information.

#### Pathway and promoter analysis

The pathway tools include gene enrichment analysis for Gene Ontology (GO) terms [21] and KEGG pathways [22] based on the hypergeometric test implemented in the GOstats package [23]. Users can select conditional testing for GO terms in order to avoid redundancy caused by the hierarchical structure of GO. In this mode, the gene list is tested for the most specific GO terms first. If significant terms are found, the genes mapping to these terms are removed before testing for the more general parent terms. As opposed to testing genes individually, the user can also perform gene set tests based on the globaltest package [24] and SAFE [25], which calculate a test statistic per GO category or KEGG pathway taking into account the expression levels of the genes. In addition to these tools running on the actual Chipster server, pathway tools running elsewhere are also offered in the Chipster client program. These include over-representation analysis with ConsensusPathDB provided by the Max-Planck Institute. ConsensusPathDB integrates functional interaction data from 20 databases covering protein-protein, metabolic, signalling and gene regulatory interaction networks [26], thus providing a powerful and combinatorial approach to pathway analysis.

The promoter analysis tools in Chipster offer a possibility to search for common sequence motifs with Weeder [27] or Cosmo [28], or search for known transcription factor binding motifs using the JASPAR matrices [29]. Transcripts are linked to the corresponding promoter sequences using RefSeq accession numbers. Promoter sequences for human, mouse, rat, drosophila and yeast are obtained from the UCSC genome browser [30].

#### miRNA analysis

The tools for miRNA analysis are applicable to most miRNA arrays including Agilent and Exiqon, as long as the data includes miRNA systematic names which Chipster uses as identifiers. The user can retrieve miRNA target genes from six different databases, run pathway enrichment analysis for the targets, and correlate miRNA expression with matching gene expression data if available.

#### aCGH data analysis

Chipster contains a comprehensive collection of tools for analysing DNA copy number data measured by aCGH. The tools include calling copy number aberrations (gains and losses) [31,32], identifying commonly aberrated regions [33], removing wavy artifacts from aCGH profiles [34], and measuring known copy number variation for the areas of interest (probes, genes or chromosomal regions) from the Database of Genomic Variants [35]. Dedicated tools are also available for clustering [36], group comparisons [37], and hypergeometric tests for enriched GO categories. These take into account the specific characteristics of aCGH data, and are therefore more suitable than the equivalent tools developed for gene expression studies. Importantly, it is also possible to integrate aCGH data with expression data to assess expression changes induced by aberrated gene copy numbers [38]. The third case study of this article demonstrates how to integrate aCGH data with gene expression data in Chipster.

As the mapping of microarray probes to their genomic coordinates is essential for all aCGH data analysis, this information can be downloaded from CanGEM, which is a public database focusing on aCGH microarray data [10]. These mappings have been obtained from probe sequences using MegaBlast [39] and are available for different builds of the human genome. Direct importing of entire data sets from CanGEM is also supported.

#### Data export to public databases and other software

In addition to analysis sessions, individual data files can also be exported from Chipster in a tabular text format at any time. These files are suitable for submission to many third-party software. Chipster can also export data in a suitable format for uploading to the ArrayExpress [8] and GEO [9] databases.

### Case studies demonstrating Chipster's analysis and visualization tools

In this section we present three case studies to illustrate the merits of some data analysis and visualization options in Chipster, such as linear modelling, alternative probe mappings, and data integration. The analysis sessions of these case studies are available for download [40] and further inspection in Chipster.

#### Using linear modelling to analyze several factors simultaneously

This case study demonstrates how to apply the linear modeling tool for a biological problem using data from the case-control study published by Lenburg [41]. They compared renal cell carcinoma tissue samples with healthy tissue from the same person, which effectively introduces a pairing structure to the data. We will model the pairing explicitly here, and also include the gender of the individual and the side of the affected kidney (left or right) as independent variables in the model. In this example we also show how to apply alternative probe mappings for Affymetrix data, in this case for the U133A arrays.

The CEL-files for the 17 samples were imported to Chipster and the quality of the data was checked using the Affymetrix-specific quality control tools including RLE and NUSE. As no deviant arrays were identified, all the arrays were retained in the dataset and normalized using the RMA method and the alternative probe mappings (altCDFs). Using altCDFs for the summarization step practically halved the number of probesets, reducing it from 22 283 to 12 133. Next the experimental setup was described using the phenodata file, which was generated during normalization. The variable corresponding to the most interesting hypothesis (here, case versus control) was coded in the group column. All the other variables of interest such as gender, side and pairing were added as new columns to the phenodata and coded with numbers. Several quality controls including PCA, NMDS and dendrogram run on the normalized data showed that the sample groups separate well from each other. Affymetrix control probes and 90% of the genes that showed the lowest coefficient of variation were removed using the tools "Search by gene name" and "Filter by CV", respectively. Chipster's filtering tools "Filter by CV" and "Filter by standard deviation" allow users to set the filtering percentage according to their needs. We used a relatively high level of stringency in this and the following case studies in order to focus on the more prominent changes in expression and to minimize false positive findings in the downstream analyses.

The genes that are differentially expressed between cases and controls, males and females, or left and right kidneys, can be analysed using tests suitable for comparing two groups. However, this is a suboptimal solution, since possible interactions between the variables can not be tested, and the effect of interest can be masked by confounding variables. To address this we used the linear modelling tool in Chipster to build a linear regression model that allows us to include all the variables in the same analysis and to take the pairing structure into account. Chipster's linear modelling tool is an implementation of the limma package [17] from the Bioconductor project. The case-control status, gender and side of the kidney were included as main effects and the patient was included as pairing. All variables were treated as categorical variables (factors). Thus, the following model was fitted

y= a+ b1*status + b2*gender + b3*side + b4*patient + e

The Benjamini and Hochberg false discovery rate (FDR) correction was applied to the p-values to adjust them for multiple comparisons.

**Figure 3:**
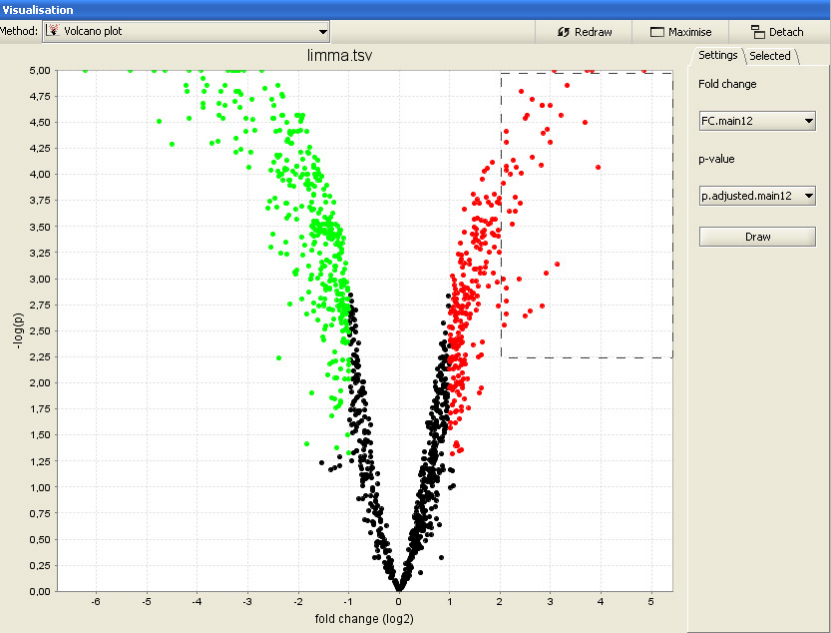
**Volcano plot of results from the linear model analysis of renal cell carcinoma**. Differential gene expression between cancer and normal samples was analyzed using linear modelling as described in the first case study of this article. The results were visualized using the interactive volcano plot of Chipster, so that the relative gene expression between cancer and normal samples was plotted on the x-axis, and the -log 10 -transformed p-values from the linear model were plotted on the y-axis. Each dot denotes one gene (probeset). The colored dots are statistically significantly (p < = 0.05) down-regulated (green) or up-regulated (red) genes. Users can select genes by drawing a box around them and create new gene lists based on these selections.

Results for the case-control comparison were visualized using the interactive volcano plot, where the x-axis contains the log2-transformed fold change values, and the y-axis contains the -log10 -transformed p-values (Figure 3). The linear modelling result was filtered for p-values using the tool "Filter using a column value". 839 genes were statistically significantly differentially expressed (p-value < 0.05) between the cases and controls, 20 genes were significant for gender comparison, and no genes became significant for the comparison between left and right kidneys. The list of genes that were up-regulated in cancer (378 genes) was enriched for GO categories Blood vessel development (GO:0001568) and Response to hypoxia (GO:0001666), as judged by the tool "Hypergeometric test for GO". Similarly, enrichment for HIF1-alpha transcription factor network and several adhesion pathways was indicated by the tool "Hypergeometric test for ConsensusPathDB". These results are consistent with the fundamental role of angiogenesis in the renal cell carcinoma pathogenesis [42].

In contrast to the analysis conducted by Lenburg et al, our results for the case-control comparison are adjusted for the other variables in the model. In other words, the results given for the case-control comparison take into account additional knowledge of the samples such as gender, side of the kidney and the patient. Lenburg et al reported 1211 UniGene clusters and 23 unannotated probesets (corresponding to 851 unique gene symbols) that had changed more than three-fold. In order to compare their result to ours, the differentially expressed genes were filtered for fold change using the tool "Filter using a column value". The list of more than three-fold changed genes (280) was then compared to that of Lenburg in the interactive Venn diagram visualization, using gene symbol as the common identifier. Only 191 genes were common to both datasets. In addition to the different analysis methodology, this difference probably reflects the use of remapped probes, which has been shown to cause up to 50% discrepancy in genes previously identified as differentially expressed [13]. Interestingly, the 89 genes detected only by Chipster included genes involved in hypoxia response (*ADM, ALDOC *and *DDIT4*), cell migration (*COL1A2*), and cell proliferation (*PDGFD*). Taken together, Chipster's linear modelling tool and alternative probe mappings enabled us to find additional genes potentially relevant to renal cell carcinoma, while keeping false positive findings due to outdated probe mappings to a minimum.

#### Analyzing a prenormalized dataset: Comparing gene expression between two populations

In this example we demonstrate how to analyse prenormalized data in Chipster by using expression data from the study by Stranger et al. [43]. They performed gene expression profiling of Epstein-Barr virus-transformed lymphoblastoid cell lines of the 270 individuals genotyped in the HapMap Consortium using Illumina's WG-6 version 1 arrays. In this example we compare gene expression in the European (CEU) and African (YRI) populations using a subset of 120 samples (parents only).

Normalized data from the Genevar site [44] of the Sanger Institute were imported to Chipster using the Import tool. The data was converted to Chipster format and the phenodata was created by using the tool "Process prenormalized". The population was indicated with numeric codes (CEU = 1, YRI = 2) in the group column of the phenodata, and the population codes (CEU and YRI) were entered in the description column in order to use them as sample labels in visualizations.

**Figure 4:**
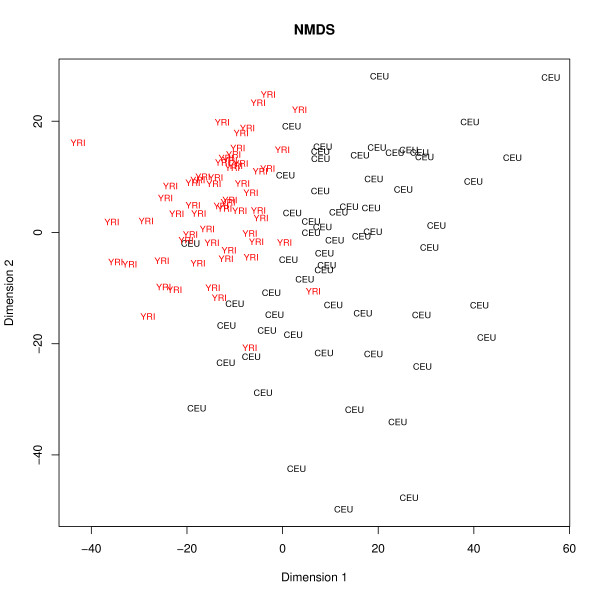
**NMDS analysis of the gene expression differences between two populations**. Normalized gene expression data for 120 European (CEU) and African (YRI) samples was obtained from the Genevar site and analyzed with Chipster's NMDS tool as described in the second case study of this article. The samples segregate to different parts of the plot, indicating that there are differences in gene expression between the populations.

Differential expression between the populations was visualized using the NMDS tool, which produces a two-dimensional map (Figure 4) based on sample dissimilarity calculated using Euclidean distance. As is instantly evident from the image, the YRI samples are more to the top-left of the image, and the CEU samples more to the bottom-right, indicating that there are differences in gene expression between the populations. The samples were also visualized in a 3-dimensional interactive scatterplot using the three most significant components from a PCA analysis. Again, it was noted that samples clearly segregated according to population, but no further sample clustering could be observed upon close examination of the data points along any axis and direction of view, suggesting that no additional underlying sample characteristics exhibited any major impact on the expression patterns.

Differentially expressed genes were analysed using the empirical Bayes test, after filtering out 95 percent of the probes that showed the lowest standard deviation. 1601 probes corresponding to 1233 known genes were statistically significantly differentially expressed between the populations at the 5% false-discovery rate. In order to gain functional insight, the differentially expressed genes were analysed for enrichment in GO categories for biological process using the tool "Hypergeometric test for GO" with default parameter settings. Interestingly, the most enriched category was immune response. The list of differentially expressed genes was further filtered on fold change using the tool "Filter using a column value". Only 75 probes corresponding to 45 known genes showed a fold change higher than 2. Taken together, it seems that gene expression differences between populations are commonplace, but most of the differences are very subtle.

#### Integrating DNA copy number and gene expression data

This third case study illustrates the integration of aCGH and mRNA data to assess expression changes induced by DNA copy number aberrations. As the aberrations typically contain also bystander genes in addition to the driving ones, integration with expression data helps to identify the potential cancer genes. We used 32 breast cancer samples with matching aCGH data [45] and expression data [46]. This is a subset of the original study containing 106 samples, because we were able to pair data only for 32 samples using the supplementary material of the referred articles. Attempts to obtain the pairing information from the original authors were also unsuccessful.

The Agilent 4x44K aCGH data was normalized using the Agilent 2-color normalization tool with normexp background correction (offset 50) and loess normalization [1]. The Affymetrix U133A expression data was GCRMA normalized [1], and 75% of the probesets with the lowest standard deviation were filtered out. Quality of the two data sets was checked with respective quality control tools, and since no deviant samples were observed, all arrays were retained. In order to enable the integration of the copy number and expression data, the Agilent probes and Affymetrix probesets were annotated with their chromosomal positions using the tool "Fetch probe positions from CanGEM" [10].

aCGH profiles typically show a wavy artefact related to their GC content. This pattern can be removed by using clinical genetics samples measured on the same array platform as calibration data [34]. We applied the tool "Smooth waves from normalized aCGH data" using a calibration dataset of mental retardation samples [47] which had been previously normalized using the same settings as described for the aCGH data above. Smoothed log ratios were then analyzed with the tool "Call copy number aberrations from aCGH data" [31,32] to detect gains and losses. The aCGH data set was studied further by identifying commonly aberrated regions [33], which showed most frequent gains in 8q and 1q. The amount of known copy number variation (CNV) within these regions was measured with the tool "Count overlapping CNVs" [35], which annotates the data with two metrics: the number of reported CNVs that overlap with the region of interest, and the proportion of base pairs that falls within the reported CNVs. These values were compared to the mean and median across the whole genome, obtained by running the tool "Calculate descriptive statistics".

In order to assess expression changes induced by DNA copy number aberrations, the aCGH and mRNA data sets were first integrated using the tool "Match copy number and expression probes", which locates the closest copy number probe for each expression probeset. It also generates a heatmap showing the two data sets organized by chromosomal position. The effect of copy number changes on mRNA expression levels was then evaluated by a permutation-based non-parametric test [38] implemented in the tool "Test for copy number induced expression changes" using the default parameter settings. Probesets with a p-value smaller than 0.05 were selected with the tool "Filter using column value". Our analysis identified 884 genes (corresponding to 1087 Affymetrix probesets) which showed copy number induced expression changes. In the original paper, Andre et al. [45] highlighted a list of 20 frequently amplified genes, 15 of which showed significant correlation between expression and copy number. Chipster detected nine of these genes: *BRF2, DDHD2, EIF4EBP1, ERBB2, ERLIN2, FGFR1, GRB7, LSM1*, and *RAB11FIP1*.

**Figure 5:**
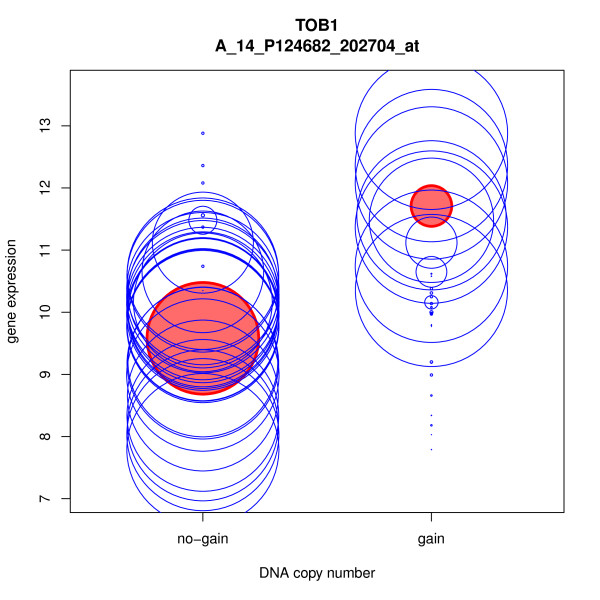
**DNA-mRNA plot illustrating copy number induced gene expression changes for *TOB1***. Copy number and gene expression data in 32 breast cancer patients was integrated, and the effect of copy number changes on mRNA expression levels was evaluated using a permutation-based non-parametric test as described in the third case study of this article. The tool "Plot copy number induced gene expression" was then used to illustrate the results for the *TOB1 *gene (Transducer of ErbB2 1). In the plot each patient is depicted as a blue circle, whose radius is proportional to the probability of the copy number call, and whose centerpoint indicates the gene expression level. The filled red circles have a radius proportional to the estimated expected call probabilities, and their centerpoint indicate the estimated mean expression for the respective call.

The resultant gene list was explored further using different filters. As *ERRB2 *is a well-known breast cancer gene, we filtered the gene list for involvement in the *ERRB2 *signaling pathway by using the tool "Extract genes from KEGG pathway". Five such genes were found, in addition to *ERBB2 *itself. We filtered the gene list also for effect size (the amount of differential gene expression induced by the copy number difference), and for the coefficient of determination, R2 (the proportion of variation in gene expression explained by copy number change). There were 25 genes for which the effect size of the DNA copy number on the gene expression was higher than two and explained over 50% of the variation in gene expression. Interestingly, one of these genes was *TOB1 *(Transducer of ErbB2 1), which has been recently implicated in breast cancer metastasis [48]. The relation between the copy number and expression data for *TOB1 *was illustrated using the tool "Plot copy number induced gene expression" (Figure 5). Taken together, these results demonstrate Chipster's ability to identify potential cancer related genes. While the integration method used by Andre et al. simply divides the samples into two groups based on DNA copy number calls, the method implemented in Chipster also takes into account the probabilities with which these calls are made (sometimes referred to as "soft calls"), which has been shown to yield improved results [38].

## Discussion

### Comparison with other microarray data analysis software

As microarrays have become a standard experimental technique for many genome-wide applications, a large number of software has been developed for their analysis. For a comprehensive review the reader is referred to the recent survey by Koschmieder et al [49]. In their thorough comparison of the currently available microarray analysis software, Chipster was the most complete in terms of analysis functionality, and it was also considered particularly user-friendly and easy to install.

Chipster differs from many other software in that it combines several important features in one package. Firstly, Chipster facilitates reproducible and collaborative research by enabling users to save the performed analysis steps as reusable, automatic workflows, which can also be shared with other users. As Chipster keeps track of the analysis, it can also produce textual reports of the steps taken. Secondly, Chipster allows the integration of different data types such as gene expression, miRNA expression and aCGH data. Thirdly, Chipster avoids the all too common "black box" approach by allowing the user to view the source code of the analysis tools. Fourthly, Chipster is a client-server system, where the client software utilizes Java Web Start technology for automatic installation and updates. The actual analysis modules, R libraries, annotations, and promoter and pathway databases are installed and updated centrally on the server side. The client-server system combines the benefits of a standalone program and web based tools: Having a full graphical user interface makes it easy to provide responsiveness and interactivity when compared to web-based applications, while the centralized approach for the analysis tools reduces the maintenance burden. It also enables the analysis jobs to benefit from the CPU and memory of central computing servers, so that the user's desktop computer is freed for other tasks. In fact Chipster's flexible architecture allows the analysis computations to be distributed to several servers, and the tool and database collection can be further expanded by connecting external Web services to the system. Finally, Chipster is open source, and new analysis tools can be easily added by using a simple tool description notation.

Among the leading freely available software that most closely resembles Chipster are Mayday [50] and MeV [51], which offer rich analysis functionality and interactive visualizations. However, Chipster differs from them both technically and functionally in many ways. In terms of functionality, the main differences lie in the quality control and normalization tools. Mayday doesn't provide array specific quality control tools, such as RLE and NUSE for Affymetrix data, and MeV includes hardly any quality control tools at all. The normalization options in both Mayday and MeV are very limited: While MeV includes basic scaling and various transformations, there are for example no specific tools for normalizing Affymetrix raw data (CEL files). Therefore users have to install additional software to handle importing and normalization tasks. Mayday includes support for CEL files, but the only normalization algorithm provided is RMA. Neither software has built-in support for Illumina arrays, nor do they offer the possibility to use remapped probe information. The tool selection for statistical testing is fairly similar in Chipster, Mayday and MeV. However, Mayday doesn't provide a linear modeling tool for the analysis of more complex multi-factorial experiments, and MeV's tool allows the analysis of only two factors without considering the interaction effects between them. On the other hand MeV offers a statistical tool for survival analysis, which is not available in either Chipster or Mayday. While the clustering options of Chipster, Mayday and MeV are very similar, the latter two provide a wider choice of classification tools. aCGH analysis is supported only by Chipster and MeV, but MeV lacks the ability to include gene expression data in an integrative analysis approach. Analysis of miRNA expression data and integration with gene expression data is only available in Chipster.

All the three software provide workflow functionality, allowing users to automate and share analysis pipelines. However, the implementation of the this functionality is very different. In Chipster the workflow is created automatically as the analysis progresses and gradually builds to a complete pipeline, which the user can edit and save at any time. In contrast, both MeV and Mayday use separate workflow building applications, which the user has to learn in addition to the main software. While the workflow panel in Chipster makes saving workflows easy, it also greatly enhances general usability: By clearly displaying the relationships between datasets it enables the user to quickly get an overview of the analysis session.

From the technical point of view both Mayday and MeV are standalone applications, while Chipster is a client-server system. Both approaches have their limitations and advantages: While standalone software doesn't need to transfer data over the network to the server, its performance is limited by the CPU and memory of the user's computer. This can be a serious limitation when performing computing intensive tasks such as hierarchical clustering, permutation-based statistical testing, or normalization of exon arrays. Taken together, the choice of software is not trivial and depends on factors like ease of installation and use, the type of data to be analyzed, the capability of the user's computer, and the availability and extent of IT infrastructure and support.

### Future development

While this paper describes Chipster's microarray data analysis functionality, it is important to remember that Chipster is a generic platform and easy to extend to other areas, even beyond bioinformatics. For example, developing the sequence analysis software Embster [52] was very fast by integrating the EMBOSS package [53] and several other analysis tools to this platform. Also adding NGS data analysis functionality to Chipster has been easy, and the current release candidate version of Chipster already contains tools for RNA-seq, miRNA-seq, ChIP-seq and methyl-seq data. It also contains a built-in interactive genome browser for viewing reads and results in their genomic context. While server based systems such as Chipster and Galaxy [7] have the advantage of being able to handle computationally heavy NGS analysis tasks, they face the challenge of transferring large amounts of data over the network. Chipster's flexible architecture has allowed us to tackle this problem efficiently using the following approaches. As described in the Implementation section of this article, a caching mechanism is used so that data is not transferred multiple times. In order to optimize data transfer even further, we are currently developing lightweight sessions which only contain links to the data stored on the server. We are also developing plugins that allow the user to connect different data servers to the system so that transfers are done directly between the servers (and when possible, skipped altogether). In terms of distributed processing we are currently working with the Hadoop map-reduce framework [54] so that large jobs can be run in the cloud.

## Conclusions

Taken together, Chipster is a user-friendly open source analysis software for microarray and other high throughput data. Its intuitive user interface brings a comprehensive collection of analysis methods within the reach of experimental biologists, enabling them to analyze and integrate different data types such as gene expression, miRNA and aCGH. The analysis tool arsenal is complemented with powerful interactive visualizations, allowing users to select datapoints and create new gene lists based on these selections. Importantly, users can save the performed analysis steps as reusable, automatic workflows. Chipster promotes collaboration at several levels: While biologists can collaborate by sharing workflows and analysis sessions, bioinformatics core facilities can also easily share their expertise with research groups by providing ready-made workflows and new analysis tool scripts. Finally, Chipster integration is an easy way for analysis method developers to provide their tool with a graphical user interface, thereby making it available for a wider group of users.

## Availability and requirements

• **Project name: **Chipster

• **Project home page: **http://chipster.csc.fi/

• **Operating system(s): **Platform independent

• **Programming language: **Java

• **Other requirements: **Java 1.6

• **License: **GNU GPL version 3

• **Any restrictions to use by non-academics: **none

## Abbreviations

aCGH: array comparative genomic hybridization; altCDF: alternative Affymetrix library file; CCA: Canonical Correspondence Analysis; ChIP-seq: chromatin immunoprecipitation sequencing; CNV: copy number variation; FDR: false discovery rate; GEO: Gene Expression Omnibus; GNU General Public License; GO: Gene Ontology; KEGG: Kyoto Encyclopedia of Genes and Genomes; KNN: k-nearest neighbor; LDA: Linear Discriminant Analysis; NGS: next generation sequencing; NMDS: Non-metric Multi-Dimensional Scaling; NUSE: Normalized Unscaled Standard Error; PCA: Principal Component Analysis; RLE: Relative Log Expression; RMA: Robust Multi-Array Average; ROTS: Reproducibility-Optimized Test Statistic; SAM: Significance Analysis of Microarrays; SOAP: Simple Object Access Protocol: SNP: single nucleotide polymorphism; SOM: self-organizing map; SVM: support vector machine.

## Authors' contributions

MAK and TH designed and implemented the Chipster software platform and participated in drafting the manuscript. JTT, MG and IS implemented the data analysis tools in Chipster and participated in drafting the manuscript. PK, MK and JK designed and implemented the graphical user interface of Chipster. EIK conceived the study, and participated in its design and coordination and drafted the manuscript. All authors read and approved the manuscript.

## Supplementary Material

Additional file 1 **This file contains a figure showing the different components of the Chipster server environment**. Click here for file
