# Supplementary material for: Chipster: user-friendly analysis software for microarray and other high-throughput data
Source: BMC Genomics. 2011 Oct 14;12:507. doi: 10.1186/1471-2164-12-507 (PMC3215701; doi:10.1186/1471-2164-12-507)
Supplement: Additional file 1 — This file contains a figure showing the different components of the Chipster server environment. [file 1471-2164-12-507-S1.PDF]

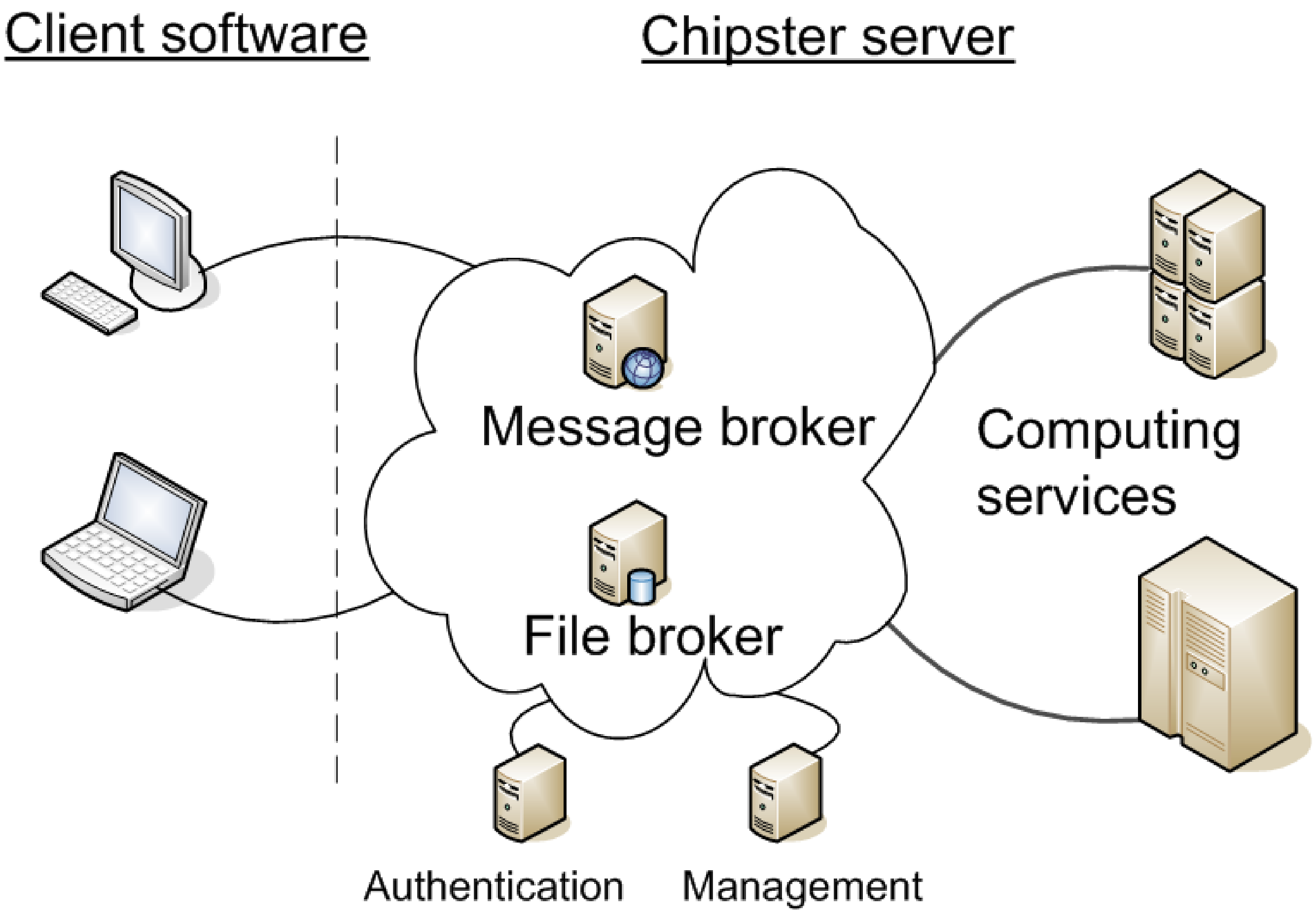

Additional file 1 - Chipster architecture

Services running on the server environment and clients running on users' workstations are connected together using brokers. Service requests and results are transferred through message brokers and related data files through file brokers. The flexible system allows services to be added on the runtime and they are automatically discovered, so that manual configuration changes are not needed.
